# Adverse infusion reactions to rituximab in systemic lupus erythematosus: a retrospective analysis

**DOI:** 10.1186/s41927-019-0082-7

**Published:** 2019-08-29

**Authors:** Ashleigh Hennessey, Joanna Lukawska, Geraldine Cambridge, David Isenberg, Maria Leandro

**Affiliations:** 1 Rheumatology Department, Royal Brisbane and Women’s Hopsital, Herston, Brisbane, Queensland 4006 Australia; 2 University of Queensland School of Medicine, Herston, Brisbane, Queensland 4006 Australia; 3 Allergy Medicine, University College London Hospitals, London, UK; 4 Centre for Rheumatology and Bloomsbury Rheumatology Unit, University College London, London, UK; 5 Division of Rheumatology, Department of Medicine, University College London Hospitals & Centre for Rheumatology, London, UK; 6 Bloomsbury, Rheumatology Unit, University College London, London, UK

**Keywords:** SLE (systemic lupus Erethematosus), Rituximab, Infusion reaction, Biologics, B cells

## Abstract

**Background:**

To undertake a retrospective review of patients with SLE who had received Rituximab in order to determine the rates and associated patient characteristics of clinically significant adverse infusion reactions.

**Methods:**

A descriptive analysis was undertaken of each infusion reaction, which was then assessed using the clinical information available to hypothesise on the possible underlying mechanism(s).

**Results:**

Records of 136 SLE patients previously treated with 481 individual infusions of Rituximab were reviewed. A total of 22 patients (17.6%) had 28 (5.8% of total infusions) documented clinically significant adverse infusion reactions. Average age at first Rituximab infusion in patients without a reaction was 37 years (range 16–73) compared with 30 years (range 18–56) in those with a reaction. A high proportion of men (18.2%) experienced an infusion reaction. Severity and type of reaction varied. 6.4% of those who had a reaction were not retreated.

**Conclusions:**

While Rituximab remains an important tool in the treatment of SLE it is important to be aware that rates of infusion reactions may be more significant in SLE than in other diseases. A prospective study is required to better characterise the reactions.

## Background

In spite of greatly improved survival rates and more effective treatments over the past 50 years, patients with Systemic Lupus Erythematosus (SLE) continue to suffer significant morbidity and mortality. Despite the successful introduction of novel biologic therapies for the management of patients with Rheumatoid Arthritis (RA) and Psoriatic Arthritis, this has not been matched in patients with SLE. The Centre for Rheumatology at University College London (UCL)/University College London Hospitals (UCLH) introduced the treatment of Rituximab (RTX) for the treatment of SLE in 2000 [1]. The majority of this first cohort, were patients who had failed to respond to and/or had suffered unacceptable side-effects from conventional therapy.

RTX is a chimeric mouse/human monoclonal antibody against the CD20 antigen which leads to immune mediated B-cell death [1] . The positive effects of RTX in patients with SLE have been described by over 20 rheumatology/nephrology units internationally in open-label trials and retrospective reviews of cohorts [2, 3], however neither of the two major randomized clinical trials EXPLORER [4] (treating patients with non-lupus nephritis) and LUNAR [5] (which focused exclusively on patients with significant renal disease) met their primary end points [3, 6]. Despite this, RTX is included in the National Health Service England Guidance for the management of SLE [7], BSR guidelines [8] and the American College of Rheumatology’s renal guidelines for use in SLE nephritis when conventional therapies have failed [9].

Our most recent detailed analysis of outcome of 115 patients with SLE treated with RTX, and followed for a minimum of one year [10], reported that 67% of patients attained full or partial remission utilizing the British Isles Lupus Assessment (BILAG) tool [11].

An important rate limiting step in the use of RTX in patients with SLE has been the highly unexpected “attrition rate” due to apparent allergic reactions occurring during infusions. This problem has frequently prevented re-treatment with RTX in patients who showed a good response to initial cycle(s) of therapy. The published rates of infusion reactions (IRR) to RTX in SLE are available from the aforementioned clinical trials but vary in range from 16.4 to 43.8% [4, 5]. The UCLH report on the 115 SLE patients who had received RTX quoted 26 adverse/infusion related reactions with a rate of 6.1% of patients, 11.6% of RTX cycles of treatment [10]. None of these reports however provide descriptions of the characteristics of the reactions.

Adverse reactions to biologics, and in particular to RTX, are complex. The extent to which individual IRRs reflect the activation of the immune system with potential cytokine release directly associated with the drug mechanism-of-action, or are true hypersensitivity reactions (and of what type) is difficult to ascertain [12, 13]. Most of the available published literature on adverse reactions to RTX focus on patients with haematological malignancies. B-cell function and number (load) can impact the rate of IRRs and severity [14–16]. There are very few publications describing patients with autoimmune rheumatic diseases, and, of those, most concern Rheumatoid Arthritis rather than SLE [17–19].

Circulating pre-existing or newly synthesised human anti-chimeric antibodies (HACA) to the drug or the cytokine profile present at the time of the infusion may also underly the triggering of adverse events. In vitro, RTX-specific IgE and Th2 cells have been demonstrated in a patient with RA who had experienced an allergic reaction to RTX [20]. HACAs do not necessarily have to be of IgE class to stimulate a clinical hypersensitivity reaction, as there have been reported cases with IgG as the reaginic class in infusion reactions associated with other biologics [21]. There have also been case reports of IRRs without detectable HACAs in a Non-Hodgkins lymphoma cohort [22].

In the non-HACA related IRR, symptoms can be caused by cytokine release syndrome (CRS), with a predominance of first dose reactions. This is a clinical event that is difficult to distinguish from true hypersensitivity reaction. CRS is associated with acute release of inflammatory cytokines (tumour necrosis factor, interferon gamma, interleukin (IL)-6, and IL-2) [23]. It is usually mild and effectively treated with reduction of infusion rate. However, CRS can also be associated with significant morbidity, due to the activation of signaling pathways resulting in a cytokine ‘storm’. The most notable example of this was reported following administration of an agonistic monoclonal antibody targeting an activatory T cell antigen (product TGN1412-anti CD28) [23, 24].

The classification of IRRs most often used in the clinic is the Modified National Cancer Institute (NCI) Common Terminology Criteria for Adverse Events Scale [25]. This is a purely descriptive scale without proposing a mechanism of the underlying reaction and therefore provides little guidance for re-treatment decision making. When this scale is applied, there is also no requirement to attribute the reaction to the particular drug being infused. In addition, any coinciding symptoms which are recorded are reported for the purpose of clinical trials and post-marketing surveillance. Whilst this is imperative for safety in a clinical trial, it does not provide any information on the immunological mechanisms underlying the reactions.

There would obviously be a clear benefit in identifying those patients most at risk of developing significant adverse reactions and understanding the precise mechanism of individual IRRs. This would allow more targeted treatment and risk assessment and therefore help guide decisions regarding retreatment of individual patients. There are now well established desensitisation protocols for patients who have a true hypersensitivity reaction to RTX, which could potentially allow retreatment with this often highly effective drug for SLE [26–29].

The aim of this study therefore was to identify the rates of clinically significant adverse IRRs to RTX, and possible associated characteristics, in the SLE cohort of patients at UCLH.

## Methods

A retrospective analysis of the complete SLE cohort at UCLH who received RTX as part of their clinical care from June 2000 to May 2016 was undertaken. Patient files and electronic records (including clinic letters and discharge summaries) were systematically examined for the presence of a clinically significant reaction (documented in) for each RTX infusion. As this was a service evaluation, as deemed by UK Health Research Authority no specific ethics approval was required.

When RTX is administered prednisolone and hydroxychloroquine is continued, however all other immunosuppressive medication is ceased. RTX was given with routine pre-medications which include methylprednisolone, an antihistamine and paracetamol. Patients were treated with two infusions of 1 g of RTX on Days 1 and 15. Each cycle of RTX therefore refers to a pair of infusions (RTXa, RTXb). For 4 patients, 4 infusions were given (RTXa, RTXb, RTXc, RTXd). An infusion refers to a single dose. Prior to 2007 it was standard of care to administer 750 mg of cyclophosphamide following the first RTX infusion. The protocol for administration is an initial infusion rate of 50 mg/hr. This is continued to 30 min and the patient is then assessed for continued stability of pulse, temperature and blood pressure, and absence of any symptoms suggesting hypersensitivity. The infusion rate is then increased to 100 mg/hr. followed by up-dosing by 50 mg/hr. until the maximum infusion rate of 400 mg/hr. is reached. On Day 15, initial infusion rate is 100 mg/hr. and increased in 100 mg/hr. increments to a maximum of 400 mg/hr. If the patient experienced an IRR during the first infusion, but which was safely managed by decreasing the flow rate or temporarily stopping the infusion and the infusion was completed, the Day 15 infusion rate was delivered using the first-dose protocol.

Details regarding the nature and timings of the reaction were recorded, as was the decision making surrounding patient management (when available). To differentiate between likely cytokine release or antibody mediated reactions, we used the following criteria: the timing of the onset of the reaction, signs and symptoms (skin flushing and nonspecific symptoms v. combination of signs/symptoms involving at least two body systems), response to treatment (able to continue infusion following simple mesures such as rate reduction or the recurrence/deterioration of symptoms on continuation) and whether treatment needed to be used to control the IRR. Clinical information including demographic, organ involvement and autoantibody specificities, details regarding subsequent infusions were recorded and when available, the decision-making process surrounding retreatment (or not) were also collected.

Using GraphPad PRISM Version 7, descriptive statistical analysis was undertaken. Mann-Whitney Rank Sum nonparametric tests were applied for continuous variables. For nominal variables Chi-squared test was used with a confidence interval set at 95%.

## Results

Records of 136 SLE patients previously treated with RTX were reviewed. 11 patients (21 cycles) were excluded due to missing information, giving a total of 481 individual infusions of RTX in 125 patients (118 females and 7 males) available for analysis. Five patients required more than two doses of RTX to achieve clinical response (2 cycles comprised of 3 infusions and 4 cycles comprised of 4 infusions).

As shown in Tables 1, 22 patients (4 male, 18 female) (17.6%) had 28 IRRs (5.8% of a total 481 RTX infusions). The average age of those with a reaction to the first infusion was 30 years (range 18–56) and in patients without a reaction was 37 years (range 16–73). Patients received between 1and 9 cycles of RTX. Most IRR occurred within the first infusion in a cycle (*n* = 7; 25%), and 19 with the second dose (67.9%) and 1 each in third and fourth infusions within a cycle (Table 2). Three patients were retreated despite having experienced a previous IRR (one patient twice), and 2/3 of these had a recurrence of clinically significant adverse infusion reactions. Of the other 19 patients with IRR, in 11 patients (50%) there were documented concerns about their reaction, but for a significant number of the patients there was no clear documentation in the patient file about the decision making concerning decision not to retreat. Most patients (86.4%), however, were not retreated.

**Table 1:** Patient characteristics

	No Reaction	Reaction	
	103	22	
Female	100 (97.1%)	18 (81.8%)	
Male	3 (2.91%)	4 (18.2%)	0.005**
Age at first dose of RTX	37 (16–73)	30 (18–56)	0.068
Cycles of RTX	1.91 (1–9)	2.18 (1–8)	0.202
ANA positive	90 (87.4%)	22 (100%)	0.066
dsDNA positive	65 (63.1%)	16 (72.7%)	0.972
No ENA pos	25 (24.3%)	6 (27.3%)	0.767
1 ENA pos	26 (25.2%)	5 (22.7%)	0.804
2 ENA pos	28 (27.2%)	8 (36.4%)	0.388
3 ENA pos	16 (15.5%)	2 (9.1%)	0.435
4 ENA pos	8 (7.8%)	1 (4.5%)	0.596
Anti-Ro positive	57 (55.3%)	12 (54.5%)	0.946
Anti-La positive	24 (23.3%)	2 (9.1%)	0.136
Anti-Sm positive	33 (32.0%)	5 (22.7%)	0.389
Anti-RNP positive	48 (46.6%)	12 (54.5%)	0.498
Caucasian	40 (38.8%)	9 (40.9%)	0.856
Afro-Caribbean	43 (41.7%)	10 (45.5%)	0.749
Asian	16 (15.5%)	2 (9.1%)	0.435
Other	4 (3.9%)	1 (4.5%)	0.886

NB. “x ENA” refer’s to the number of antibodies to extractible nuclear antigen’s detectable in an individual patient

**Table 2:** Cycle number and reaction rate

RTXa	7 (25%)
RTXb	19 (67.9%)
RTXc	1 (3.6%)
RTXd	1 (3.6%)

NB. 22 patients had 28 reactions

RTXa first infusion of the cycle, RTXb second, RTXc third and RTXd fourth

When considering all reactions, IRRs ranged in severity from mild to severe (Table 3), however, when using the NCI modified severity criteria 35.7% of the IRR could not be classified using this system due to lack of sufficient clinical information. We therefore additionally considered IRR in terms of clinical management including whether they required complete cessation of the infusion and/or admission to hospital. More IRR were then able to be classified (Table 4).

**Table 3:** Severity of reaction by modified NCI scale

Grade 1A	Cutaneous rash, flushing, generalised pruritus	5 (17.9%)
Grade 1B	1A plus, back pain and or HTN	2 (7.1%)
Grade 2	Urticaria, nausea/vomiting, throat tightness, *Asymptomatic* bronchospasm, chest tightness	3 (10.7%)
Grade 3	Symptomatic bronchospasm, dysponea, hypoxia, wheeze	7 (25%)
Grade 4	Anaphylaxis, hypotension	0
Grade 5	Death	1 (3.6%)
Unclassified	due to lack of information available to stratify severity	4 (14.3%)
Delayed	adverse event occurred after 48 h of infusion	6 (21.4%)

**Table 4:** Management of adverse reactions

Management	No.	% of total reactions	Retreated?
Able to resume/complete infusion	8	28.6%	Y - 1 (2 further cycles *with reaction*)
Had to cease infusion	7	25.0%	Y - 1 (2 further cycles)
Hospital Admission > 24 h	3	10.7%	Y - 1 (1 further cycle) reaction)
Delayed reaction	5	17.9%	Y - 1 (1 further cycle)
Unclassified^a^	4	14.3%	N
Death	1	3.6%	n/a

^a^Due to lack of clinical data

Available clinical information was then examined for evidence of possible mechanisms underlying the IRR by a Allergy Medicine Specialist (JL) (Table 5). We used the timing of the onset, signs and symptoms as well as the response to subsequent IRR management to differentiate between potential causes. The reactions were initially classified as ‘immediate’ and ‘delayed’. Immediate reactions were the most common reaction type and were then broadly classified as likely immune mediated and likely non-immune mediated. Likely immune reactions were then classified as: likely cytokine mediated, likely immunoglobulin mediated and bone pain. Delayed IRRs were classified as Early delayed (24 to 48 h) and Late delayed (> 48 h) when information was available. One patient had a severe reaction attributed to cyclophosphamide and was excluded from analysis. Examples of “unlikely immune mediated” include a patient who was pyrexial during the infusion but was ultimately diagnosed with a UTI, another had isolated hypertension associated with the infusion. We included “bone pain” as a specific category in likely immune reactions.

**Table 5:** Proposed Mechanism of Adverse Reactions

	Immediate Hypersensitivity				Delayed Hypersensitivity		Total
Cycle Number	Unlikely immune mediated	Likely cytokine release	Likely immunoglobulin mediated	Bone pain	Early (24–48 h)	Late (> 48 h)	
1	1	2	3	2	1	1	10
2	2	3	1			3	9
3		2	1			1	4
4							
5							
6	1						1
Total	4	7	5	2	1	5	24

Based on review of the clinical description of the reaction by Clinical Allergist

4 reactions were excluded from this analysis; 1 death as likely CYC induced ARDS, and 3 due to lack of data

## Discussion

The primary objective of this study was to examine the rate of clinically significant adverse reactions to RTX in a retrospective analysis of a large single center cohort of SLE patients. We also examined the cohort to determine any significant patient characteristics in those that experienced IRRs to RTX to identify clinical risk factors for reaction. Despite the constraints of a retrospective review and consequent lack of clinical data, the rate was calculated at 17.6% of SLE patients and 5.8% of total infusions. This is the first time a detailed description of IRR in a consecutive patient cohort has been explored with the addition of further patients since the earlier published safety data. [10] This rate is similar to published IRR rates in patients with Lupus nephritis [5]. In a small study looking at the safety of rapid infusion of rituximab the quoted rates of reaction was overall 18.5% in 54 patients with autoimmune disease but only 6 of these had SLE [30]. In another study 9.4% was quoted for IRR to RTX in primary autoimmune diseases (comprising of a total of 74 patients with RA, Sjögrens and ITP) [31]. The 17.6% reaction rate is high when considering that these were all clinically significant reactions with 86.3% of these patients not retreated, compared to 13.9% patients overall who only received one cycle implying that the reactions influenced clinical decisions not to retreat.

Whilst there may have been other clinical reasons for avoiding RTX for each individual patient, given the proven efficacy of RTX in this same cohort with responder rates (partial and complete combined) of 67% [10], it is possible that they were not retreated due to no further clinical need, but it would be important to be able to provide a framework or clarity on the mechanism of reaction and also safety for retreatment if it should be required.

We have shown that 35.7% of IRR (10/28) in our SLE cohort were severe enough to require cessation of infusion or hospital admission. One patient died in the cohort, although in this instance this was likely due to the co-administered cyclophosphamide. The rate of grade 3–4 reactions in a non-SLE cohort is estimated at 10% [22, 25], in our study this was 25% (7/28). We do acknowledge that the design of the retrospective review may bias the data to appear more significant, however, our results do appear to suggest that SLE patients may be at a higher risk of IRRs when compared with patients with other autoimmune diseases. Probably due to the differences in B cell biology in patients with SLE. A subsequent study in a subgroup of 57 of these patients the presence of HACAs has been associated with the occurrence of infusion reactions to rituximab [32].

We did not attempt to qualify “back pain” in terms of mechanism other than “likely immune mediated”. Back pain features in RTX adverse reaction classification (Grade 1) [25] but it is completely absent from other classifications of allergic reactions [33]. Interestingly it is reported in the pivotal case report of cytokine release syndrome [24], so despite the pathophysiology being poorly understood it is a well-recognized phenomenon. In our patients it was not associated with other significant symptoms, except for fever in one patient.

Another interesting finding was the relatively high proportion of males (4/7 compared with 18/118) who experienced an IRR. This is consistent with results published in the haematology and oncology literature [25, 27]. When considering other allergic reactions there is no significant sex differences in the rates of IgE mediated drug (penicillin) reactions [34]. However presentations for allergic disease are female predominant in adults [35], so the reasons for the phenomena observed here are not clear although possibly due to higher cytokine release.

We also noted that most reactions occurred with the second infusion, which contrasts with available literature in the SLE clinical trials and other diseases, where overwhelmingly the first infusion confers the highest risk [4] [5]. This may well be due to under-reporting of milder IRRs in our cohort and being a retrospective study.

It was also of interest that there was little difference in terms of the cycle number in the IRR vs No IRR groups. As most patients who had an infusion reaction were not retreated, this highlights the need for constant vigilance for IRR which needs to be maintained over each infusion during additional cycles of RTX.

An unmet clinical imperative is to be able to establish the nature of the IRR in individual patients. In those with a demonstrated allergic mechanism for a reaction, slowing the infusion rate, which is often the 1st line treatment of IRR in the available protocols, is not sufficient [26, 27, 36]. The advent of desensitisation for biologics, which has been demonstrated as effective and safe [26] may potentially allow more patients to continue to benefit from RTX in spite of suffering an IRR, especially in those with few other therapeutic options. In this cohort there are an estimated 20.8% (5/24) of reactions proposed to be immunoglobulin (IgE or IgG) mediated and therefore possibly amenable to desensitisation.

There are also significant limitations of a retrospective review, the most significant of which are lack of data available with regards to concurrent medications, timing of the pre-medications, timing of the reaction, previous drug allergies and reliable history of atopy. These would be important things to capture in future prospective studies.

## Conclusion

This retrospective review of SLE patients who received RTX reports a detailed exploration of adverse reactions. Rates of IRRs to RTX appear to be more significant than in SLE than in other disease cohorts. They can occur at any time during individual treatments and during different cycles and are likely to have several different underlying aetiologies. In our cohort reactions were more common in men. Further prospective studies are needed to establish relevant mechanisms, and hence best practice for treatment and prevention.

## Data Availability

The datasets used and/or analysed during the current study are available from the corresponding author on reasonable request.

## Abbreviations

BSR: British Society of Rheumatology
CRS: Cytokine release syndrome
HACA: Human anti-chimeric antibodies
IRR: Infusion reactions
RTX: Rituximab
SLE: Systemic Lupus Erythematosus
UCLH: University College London Hopsital
UTI: Urinary tract infection
